# Supplementary material for: Root treatment with oxathiapiprolin, benthiavalicarb or their mixture provides prolonged systemic protection against oomycete foliar pathogens
Source: PLoS One. 2020 Jan 13;15(1):e0227556. doi: 10.1371/journal.pone.0227556 (PMC6957293; doi:10.1371/journal.pone.0227556)
Supplement: Supplementary file 1 [file pone.0227556.s001.docx]

Root treatment with oxathiapiprolin, benthiavalivarb or their mixture provides prolonged systemic protection against oomycete foliar pathogens

Yigal Cohen

The Mina & Everard Goodman Faculty of Life Sciences, Bar-Ilan University, Ramat-Gan 5290002, Israel

Professor Yigal Cohen
Faculty of Life Sciences
Bar Ilan University
Ramat Gan
Israel
Lab 972 3 5318 251
ycohen@biu.ac.il

— Accept with major revision.
— Corrections indicated
— Copy attached.

~~Accept with major corrections~~ and revision. English is poor.
~~Drafting is poor.~~

Abstract

Oxathiapiprolin is a fungicide effective against downy mildews and late blight. To avoid resistance, oxathiapiprolin is applied in mixtures with other fungicide having a different mode of action. Here I show that a single application of oxathiapiprolin, benthiavalicarb, or their mixture (3+7, w/w) to the root system of nursery plants growing in multi-cell trays provides prolonged systemic protection against late blight and downy mildews in growth chambers and the field. Soil application of 1mg active ingredient per plant provided durable protection of up to four weeks in tomato against late blight, cucumber against downy mildew and basil against downy mildew. Not only does the mixture of oxathiapiprolin and benthiavalicarb provide excellent systemic control of these diseases but also mutual protection against resistance towards both oxathiapiprolin and benthiavalicarb.

Key Words: Basil; CAA-fungicides; Cucumber; Orondis; Oxysterol binding proteins; *Phytophthora infestans; Pseudoperonospora cubensis; Peronospora belbahrii*; Resistance risk; Synergy; Tomato; Zorvec.

Introduction

Oxathiapiprolin is a piperidinyl-thiazole-isoxazoline fungicide (FRAC code 49) [1, 2] with extremely high activity against plant pathogenic oomycetes [3-11] except Pythium species [2]. Oxathiapiprolin is effective when applied to foliage or to the root system [12,13] Oxathiapiprolin is effective when applied preventatively or curatively and shows translaminar and acropetal systemic translocation [2, 3, 5,11].

The molecular target of oxathiapiprolin is the oxysterol binding protein (OSBP) [2,14], a member of the OSBP- related proteins family of lipid transfer proteins [15]. The target proteins of oxathiapiprolin in P. *capsici* and P. *cubensis* genome were annotated, but their function are unknown [5, 16].

Oxathiapiprolin is a high-risk fungicide [5, 12, 14, 16], which requires careful use in the field to avoid buildup of resistant mutant isolates. FRAC (http://www.frac.info/) considered the resistance risk of oxathiapiprolin as medium to high and, which requires careful resistance management [17, 18].

Available oxathiapiprolin-based fungicides include mixtures with chlorothalonil (Orondis-Opti), mandipropamid (Orondis-Ultra), azoxystrobin, mefenoxam (Orondis-Gold), famoxadone (Zorvec Encantia) and benthiavalicarb (Zorvec-Endavia). We have recently reported on synergistic efficacy of the four former mixtures against late blight in tomato [9] and downy mildew in cucumber [4].

In the present study, I examined the efficacy of oxathiapiprolin+benthiavalicarb (Zorvec-Endavia [ZE]) in controlling foliar oomycete pathogens. Because both compounds are systemic, I also examined the systemic protective efficacy of ZE when applied to the root system by soil drench.

Bentiavalicarb (together with mandipropamide, dimethomorph, flumorph and iprovalicarb) belongs to the carboxylic acid amides (CAA) group of fungicides (FRAC code 40). Their mode of action involves inhibition of cell wall synthesis of oomycetes by blocking the activity of cellulose synthase *Ces*3A of the pathogen. They are effective against *Phytophthora infestans* [19] , *Plasmopara viticola* [20, 21], *Pseudoperonospora cubensis* [22] and *Bremia lactucae* [23]. Resistance against CAAs occurs in field populations of *P. viticola* [21] and *P. cubensis* [24] but not in *P. infestans* or *B. lactucae* [25]. With *P. infestans*, we were able to artificially

mutate sporangia for stable resistance to the phenylamide fungicide mefenoxam but failed to select mutants with stable resistance against CAAs [26], suggesting a low risk of resistance developing in this pathogen in field populations against CAAs.

Soil treatment with oxathiapiprolin was reported to be effective against soil borne oomycete pathogens, e.g., black shank in tobacco caused by *Phytophthora nicotianae* [12] and root rots in citrus caused by Phytophthora species [13]. In our recent papers, we reported that root treatment with oxathiapiprolin and oxathiapiprolin+mefenoxam were effective in controlling downy mildew in cucumber [4] and late blight in tomato [9]. The half-life time of oxathiapiprolin in soil is about a week [27, 28].

The objective of the present research was to examine the efficacy of root treatment with oxathiapiprolin, benthiavalicarb or their mixture oxathiapiprolin+benthiavalicarb (Zorvec Endavia) in controlling downy mildew in cucumber, downy mildew in basil and late blight in tomato in growth chambers and the field. The unique finding of this study is that a single application of such fungicides to the root system of very young plants provides them with prolonged systemic protection against disease.

Materials and Methods

Fungicides. Oxathiapiprolin (OXPT) 100OD (oil dispersion) was a gift from DuPont, France. Benthiavalicarb (BENT) 98% technical grade was a gift from Syngenta Crop Protection, Switzerland. The mixture of OXPT+BENT (Zorvec Endavia 100SC=ZE, 30g oxathiapiprolin and 70g benthiavalicarb per Kg) was a gift from Agrochem Ltd (Petah Tikva, Israel). The fungicides (except BENT) were suspended in water and diluted to a series of 10-fold concentration suspensions from 0.001 to 1 mg ai per ml. BENT was dissolved in DMSO (100 mg/10 ml) and then suspended in water. For the dual mixtures, an indicated dose represents the combined doses of both ingredients.

Plants. Cucumber (*cv* Nadyojni, own production) and tomato (*cv* Roter Gnom, deterministic growth type, a gift from Syngenta, Switzerland, or *cv* 129, non- deterministic type, a gift from Hazera Seeds, Israel). Plants were grown from seeds in the greenhouse, in either 250 ml pots filled with peat: perlite (10:1, v/v) soil mixture, one plant per pot or in multi-cell trays (cell size 5x5 cm, Hishtil, Petah Tikva, Israel) filled with the same soil mixture. Cucumber plants were

used at the 1-leaf stage, unless stated otherwise. Tomato plants were used at the 3-leaf stage, unless stated otherwise. Basil plants (*cv* Perri, a gift from Volcani Center, Newe Yaar, Israel) were grown in multi-cell trays (cell size 2.5x2.5 cm, Hishtil, Petah Tikva, Israel). Plants that grew in multi-cell trays were transplanted to 250 ml pots or to the field as indicated below.

Root treatment. A single aliquot of OXPT, BENT or ZE was applied to the root system (soil drench) of cucumber, tomato or basil plants. Tomato plants growing in 250 ml pots were treated at the 5, 8, 10 or 12-leaf stage. Cucumber plants growing in similar pots were treated at 1, 2, 3 or 6-leaf stage. Basil plants growing 250 ml pots were treated at the eight or 10-leaf stage. Plants growing in multi-cell trays were treated at the 1-leaf stage (cucumber), 2-leaf stage (basil) or 3-leaf stage (tomato).

Root treatment was performed by pipetting 1 ml fungicide suspension of 10, 100 or 1000-ppm a.i. (0.01, 0.1 or 1 mg ai/plant) onto the soil surface around the stem base of each plant, growing in 250 ml pots or in multi-cell trays (Fig.1). In some experiments, 0.3, 0.7 or 1 ml of 10, 100 or 1000-ppm a.i. of OXPT, BENT or ZE, respectively, were applied to the root system of each plant. At 1h and 5h after treatment, 2 ml water were added to the soil surface of each treated plant to facilitate the uptake of the fungicide(s) by the root system.

Pathogens and inoculation. Isolate 260 of *Pseudoperonospora cubensis* (collected from cucumbers in Ahituv, Israel in May 2018) was used for inoculation of cucumber in growth chamber studies. This isolate is resistant to mefenoxam (MFX) and belongs to the A1 mating type. Isolate 164 of *Phytophthora infestans* (collected in March 2016 from potato at Nirim, Western Negev, Israel) was used for inoculation of tomato plants in growth chambers. This isolate is resistant to MFX and belongs to genotype 23_A1. Isolate Knafo 3 of *Peronospora belbahrii* was used to inoculate basil plants in growth chamber studies. This isolate was collected in 2013 at Ein Tamar, Southern Jordan valley, Israel; it is resistant to MFX.

Growth chamber experiments. Root-treated and control untreated plants growing in 250 ml pots were spray-inoculated with spore suspension (5000 sporangia/ml) of their respective pathogen at various time intervals after soil drench (see below). Disease records were taken at 7dpi, unless stated otherwise. In cucumber, the number of downy mildew lesions produced in each plant (or leaf area infected) was recorded [4]. In tomato, the proportion of blighted leaf area was visually estimated [9]. In basil, the proportion of infected leaf area was estimated as described before [29].

Field experiments. Experiments were conducted at Bar-Ilan University Farm (32Ê 04' 2.40" N, 34Ê 50' 19.79" E) in 50x6 m net houses covered with white, 50 mesh, insect-proof plastic screens. Root-treated and control untreated cucumber and basil plants were transplanted to the field, at one or more days after treatment, where they were subjected to natural infection with *P. cubensis* or *P. belbahrii.* Disease records were taken upon appearance of the first symptoms of downy mildew and continued at intervals of 1-4 days for 3-4 weeks after transplantation.

Data Analysis. Eight growth chamber experiments were conducted with 4-20 replicate plants per treatment. Effective doses (ED90 values, the dose of fungicide required to inhibit disease by 90% relative to control) were derived from log-probit regression curves using SPSS software. SF (synergy factor) was calculated using the Abbott or Wadley formulae as described before [30, 31]. T-test was performed using SPSS software to determine significant differences between means at $\alpha = 0.05$.

Six field experiments were performed with cucumber and six field experiments with basil, with one or two replicate plots (0.6 x 1.2m each) per treatment and 8-10 plants per plot. Percent season-long protection for each fungicide was calculated as the ratio between the AUDPC (area under disease progress curve) of the treated plants and the AUDPC of the control fungicide-free plants [4, 29].

The synergistic interaction between oxathiapiprolin and benthiavalicarb was calculated according to Abbot or Wadley as described before [30, 31].

Results

Growth chamber experiments

Tomato late blight

Exp. 1. Six leaf tomato plants (*cv* Hazera 129) growing in 250 ml pots were root-treated by soil-drench with 0.5 or 1 mg a.i. of BENT, OXPT or ZE. Plants were inoculated a day later with *P. infestans* and assessed for disease development at 8 dpi (days post inoculation). MIC (minimal inhibitory concentration) values for BENT, OXPT and ZE were 1, > 1 and 0.5 mg a.i./plant, respectively (Fig.2 A). ZE of 0.5 mg ai/plant provided a significantly better control of the blight than the other fungicides at the same dose thus exhibiting a synergy factor (SF) of 1.2 (Abbott). The appearance of the plants at 8 dpi is shown in Fig. 2 B-D.

Exp. 2. ZE is a mixture composed of OXPT and BENT at a ratio of 3:7. The following experiment was performed to learn if synergistic interaction occurs between the components of this mixture. The root system of 6-leaf tomato plants was treated with BENT of 0.007, 0.07, 0.7; OXPT of 0.003, 0.03, 0.3, or ZE of 0.01, 0.1, 1 mg a.i/ plant. Plants were inoculated with *P. infestans* at 2 days after treatment and scored for late blight symptoms at 8 dpi. The results are given in Fig.3. Increased doses of either fungicide increased disease control (Fig. 3 A, B). ZE was significantly the most effective fungicide. Probit analysis revealed ED90 values of 0.222, 0.164 and 0.04 mg a.i. for BENT, OXPT and ZE, respectively. Based on these values the expected ED90 of ZE is 0.200, thus providing a high SF value (Wadley) of 50.

Exp. 3. Five leaf tomato plants growing in 250 ml pots (n=40) were soil-drenched with 0.3, 0.7 or 1 mg a.i. of OXPT, BENT and ZE, respectively. They were inoculated with *P. infestans* at 3 DAT and scored at 7 dpi. The results are presented in Fig. 4 A-E. The data show a significantly better control of late blight by ZE compared to BENT or OXPT (Fig. 4A). Percent protection provided by OXPT, BENT and ZE was 87, 93 and 100%, respectively.

Exp. 4. The long-term protection against late blight by root treatment with ZE was examined as follows: 3-leaf tomato plants growing in multi-cell trays were treated by a soil drench with ZE of 0.5 or 1 mg a.i per plant. They were transferred to 250 ml pots a day later and were taken for inoculation (n=10) at various time intervals after treatment. The results presented in Fig. 5 show complete control of the disease up to 42 DAT. At 54 DAT, the lower dose of ZE was partially effective, while the higher dose was fully effective. At 81 DAT, when plants developed 20 leaves, ZE of 0.5 and 1 mg a.i. provided 80 and 85 % protection, respectively (Fig. 5 A, B).

Cucumber downy mildew

Exp. 1. The root system of 3-leaf cucumber plants growing in 250 ml pots were treated with 1 mg a.i. of BENT, OXPT or ZE. Plants were inoculated with sporangia of *P. cubensis* at the 5-leaf stage and scored for disease symptoms at 14 dpi. The results presented in Fig. 6 A show that BENT provided partial, though significant, protection relative to untreated plants, whereas OXPT and ZE provided complete control of the disease.

Exp. 2. One-leaf cucumber plants growing in multi-cell trays were soil drenched (n=12) with 0.01, 0.1 or 1 mg a.i. of BENT, OXPT or ZE. Plants were inoculated with *P. cubensis* at 3

DAT. Disease records, as taken at 7 dpi, are shown in Fig. 6 B. BENT, OXPT and ZE of 0.01 mg a.i./plant provided 39, 75 and 100% protection against the disease, respectively with SF (Abbott) of 1.18. The results show a significantly higher efficacy of ZE compared to OXPT, and of OXPT compared to BENT.

Basil downy mildew

Exp. 1. Twelve-leaf basil plants growing in 250 ml pots were treated (n=6) with 1 mg a.i. of OXPT or ZE, inoculated with sporangia of *P. belbahrii* two days later and evaluated for disease development at 12 dpi. Percent sporoulating leaf area in control plants was 68.3±7.5. OXPT provided 59% control of the disease, whereas ZE provided 100% control of the disease.

Exp. 2. Two-leaf basil plants growing in multi-cell trays were treated (n=20) with ZE of 0.25, 0.5 or 1 mg a.i. per plant applied to the root system. They were transferred to 250 ml pots and inoculated at 7 DAT. Percent leaf area sporoulating at 11 dpi in control-inoculated plants was 59.8±18.4 percentage, whereas none of the treated plants showed any sporulation of *P. belbahrii*, regardless of the ZE dose applied.

Field Experiments

Cucumber downy mildew

Exp. 1. The root system of 2-leaf cucumber plants growing in 250 ml pots was treated (n=40) with ZE of 0.01, 0.1 or 1 mg a.i. per plant. Untreated plants served as control. Plants were transplanted to the field at 1DAT (14.2.2019) and scored for downy mildew development for 4 weeks. Disease progress with time is shown in Fig. 7 A and AUDPC in Fig.7 B. Season-long protection provided by ZE of 0.01, 0.1 and 1 mg a.i./plant was 26, 53 and 100%, respectively (Fig. 7 C).

Exp. 2. The root system of 1-leaf cucumber plants growing in 250 ml pots was treated with 1mg a.i. per plant of BENT, OXPT or ZE and transplanted to the field at 13 DAT (28.4.2019). Downy mildew lesions appeared in control plots at 40 DAT, as against 52 DAT in BENT-treated plots. No disease was seen in OXPT or ZE treated plots until 54 DAT (Fig. 7 A). Season-long protection provided by BENT, OXPT and ZE was 84, 100 and 100%, respectively (Fig. 7 F). Minor disease symptoms developed in ZE-treated plots at 56 DAT.

Exp. 3. The root system of 1-leaf cucumber plants growing in multi-cell trays were treated with 1 mg a.i. per plant of OXPT, BENT or ZE and transplanted to the field one day later (12.5.2019). First lesions of downy mildew were seen in control plots at 15 DAT. Disease progress with time is shown in Fig.8 A and AUDPC in Fig. 8 B. By the end of the experiment, the plants had 12-14 leaves and carried a mean of 1350±212 lesions per plant. All three fungicides suppressed disease development in a significant manner (Fig.8 A). BENT, OXPT and |ZE provided 89, 98 and 99% long season protection, respectively (Fig. 8 C).

Exp. 4. The root system of 1-leaf cucumber plants growing in multi-cell trays were treated with 1mg a.i. per plant of OXPT, BENT or ZE and transplanted to the field four days later (11.6.2019). Disease progress with time is shown in Fig.8 D, and AUDPC and season-long protection values are presented in Figs. 8 E and 8 F, respectively. Final disease score in the control plots was significantly higher than in the treated plots (40, 5 and 10% in BENT, OXPT, and ZE treated plots, respectively) (Fig. 8 D). Final disease scores in OXPT and ZE treated plots were significantly lower than in BENT treated plots, but did not differ significantly among themselves. ZE was synergistically effective, with SF values (Abbott) of 1, 1, 1.03, 1.02, 1.08 and 1.06 at 10, 13, 15, 17, 20 and 22 DAT, respectively.

Exp. 5. Cucumber plants at their 1-leaf stage were soil-drench with 1ml suspension containing 0.01, 0.1 or 1 mg a.i. of OXPT, BENT or ZE (n=10). Plant were transplanted to the field after one day (11.7.2019) and disease progress was followed for 24 days. The results are presented in Fig.9 A-C. First disease lesions appeared at 15 DAT with a mean of 100 lesions colonizing each control plant. Within 9 days, a mean of 800 lesions developed on each control plant, which bear 12 leaves (Fig.9 A). All fungicides suppressed disease development in a significant manner regardless of the dose used (except OXPT of 0.01 mg ai/plant) (Fig. 9 A). AUDPC values are shown in Fig.9 B. Season-long protection provided by OXPT, BENT and ZE ranged between 59-100, 83-97 and 81-95 %, respectively (Fig.9 C).

Exp.6. Cucumber plants at their 1-leaf stage were soil-drench with 1ml suspension containing 0.01, 0.1 or 1 mg a.i. of OXPT, BENT or ZE (n=15). Plant were transplanted to the field at 4 DAT (1.8.2019). Disease records were taken from 12 to 23 DAT. All fungicides suppressed disease development in a significant manner regardless of the dose used (Fig. 9 D). AUDPC and season-long protection data are shown in Fig. 9 E and F, respectively. The appearance of
