# Supplementary material for: Root treatment with oxathiapiprolin, benthiavalicarb or their mixture provides prolonged systemic protection against oomycete foliar pathogens
Source: PLoS One. 2020 Jan 13;15(1):e0227556. doi: 10.1371/journal.pone.0227556 (PMC6957293; doi:10.1371/journal.pone.0227556)
Supplement: Attachment — Submitted filename: Response to Reviewers Comments ZE paper.docx [file pone.0227556.s002.docx]

Response to Reviewers Comments, ZE paper

Reviewer #1: While the scientific method performed in the paper is sound, I believe significant editing for clarity/cohesiveness needs to be done before the paper is accepted.

There are many small paragraphs that I believe could be put together to form a more coherent narrative. Paragraphs were joined as much a s possible.

When referring to a disease (i.e. downy mildew of basil) please also put the pathogens scientific name in parenthesis after the disease. This way, plant pathologists working with related, but not identical, organisms will know what organism the author refers to. Names of the pathogens were added.

Do not use "I" and "we", keep the writing the past tense and third person when possible. i.e. instead of "In our recent papers, we..." use "Prior research has shown..." or "the author" instead of "I". Done.

Benthiavalicarb is misspelled in the title. Corrected.

I believe the authors grouped experiments by organism to try and clarify what they did, however, I found this to be difficult to follow. I think grouping organisms by experiment would shorten the paper and provide additional clarity. Many of the experiments were identical, but performed on different plant pathogens/host combinations. I see no benefit in reiterating the method used, if it was identical between basil, tomato and their associated pathogens, for example. Grouping by organism seems to me better than other groupings. There are small, but significant differences between experiments done with the three organisms used in this study. I therefore incline to leaves the grouping as is.

This and more, the small but significant differences between experiments obliged me to start describing the Results of each experiment with a very brief M+M description of each experiment. In the M+M section I have denoted the comments features of all experiments done with each specific organism.
When referring to sporangia density used, please use scientific number format. i.e. 5000 sporangia should be 5x10^3. Some experiments did not have a sporangiat.l concentration stated, that too should be fixed. All experiments were done with a fixed concentration o 5x10^3^/ml. This is indicated in the text.

I think the authors using mefenoxam resistant isolates was a great choice. It adds to the importance of their work using alternate chemistries.I am glad to read this.

Overall, I want to commend the author for the extensive work they did. It was obviously a large and robust project and would make a great addition to expand the literature on Oxathiapiprolin/Benthiavalicarb sensitivity in oomycetes, once the grammatical and clarity/cohesiveness issues are addressed. This is a very nice comment.

i would be happy to review this paper again, once these issues are addressed.

Reviewer #2: Please create for experiments conducted in growth chamber and fields
Expt# Crop diseases Fungicides tested Application rate of fungicides. The rates ued there are not applicable to this paper. I used here injections of 1 ml fungicide per cell per plant. I see no way to transform it to kg ai fungicide per hectare.

Corrections in the attached file. Corrections were adopted.
